# Aerosolized prostaglandin E_1_ improves oxygenation during one-lung ventilation: A dose-finding randomized controlled trial^☆^

**DOI:** 10.1016/j.clinsp.2026.101203

**Published:** 2026-10-15

**Authors:** Pengxin Li, Xinyang Hu, Pengyi Li, Lingxi Xing, Qiang Wang, Lianbing Gu, Chunpei Wu

**Affiliations:** Department of Anesthesiology, Jiangsu Cancer Hospital & Jiangsu Institute of Cancer Research & The Affiliated Cancer Hospital of Nanjing Medical University, Nanjing, Jiangsu, China

**Keywords:** Alprostadil, One-lung ventilation, Hypoxia, Lung injury, Esophageal neoplasms

## Abstract

**Objectives:**

This study aimed to investigate the effects of varying doses of aerosolized Prostaglandin E_1_ (PGE_1_) on oxygenation and intrapulmonary shunt fraction (Qs/Qt) during One-Lung Ventilation (OLV) under 60% Fraction of Inspired Oxygen (FiO_2_). Secondary objectives included evaluating the impact on intraoperative hypoxemia, postoperative Clinical Pulmonary Infection Score (CPIS), and Postoperative Pulmonary Complications (PPCs).

**Design:**

A prospective, single center, randomized, double-blind, controlled trial.

**Setting:**

The study was conducted at a single academic medical center.

**Participants:**

A total of 160 patients aged 65‒84 years, with American Society of Anesthesiologists (ASA) physical status II or III and Body Mass Index (BMI) < 30 kg/m^2^, undergoing elective esophageal cancer surgery were enrolled. Patients with certain comorbidities, respiratory diseases, or contraindications to PGE_1_ were excluded.

**Interventions:**

Patients were randomly assigned to one of four groups: Group L ‒ Received 0.1 μg/kg PGE_1_ nebulization; Group M ‒ Received 0.2 μg/kg PGE_1_ nebulization; Group H ‒ Received 0.3 μg/kg PGE_1_ nebulization; Group C (control) ‒ Received normal saline nebulization. Nebulization was performed for 10-minutes via a modified breathing circuit into the ventilated lung before initiating OLV.

**Measurements:**

Arterial blood gas parameters (arterial partial pressure of oxygen, PaO_2_; arterial oxygen saturation, SaO_2_; arterial partial pressure of carbon dioxide, PaCO_2_), hemodynamic variables (Mean Arterial Pressure [MAP]; Heart Rate [HR]), respiratory mechanics (Peak airway pressure [Ppeak]; dynamic lung compliance [Cdyn]), PaO_2_/FiO_2_ ratio, and Qs/Qt were recorded at multiple time points during OLV. The incidence of intraoperative hypoxemia and hypotension, PPCs within 7-days, and CPIS on postoperative day-2 were also assessed.

**Main Results:**

All PGE_1_ groups showed significantly improved PaO_2_, SaO_2_, and PaO_2_/FiO_2_ ratio, and reduced Qs/Qt during the first 30-minutes of OLV compared to the control group. The High-dose group (H) demonstrated the greatest improvement in oxygenation. The incidence of intraoperative hypoxemia [Group L: 0/40 (0%), Group M: 0/40 (0%), Group H: 0/40 (0%), Group C: 3/40 (7.5%)] and hypotension did not differ significantly among groups; however, the hypoxemia finding was exploratory as the study was not powered for this endpoint. No significant differences were observed in PPCs incidence among groups, but group H was associated with a lower CPIS on postoperative day-2 compared to group C. Hemodynamic changes were transient and clinically manageable.

**Conclusions:**

Nebulized PGE_1_ at doses of 0.1–0.3 μg/kg improves oxygenation and reduces Qs/Qt during OLV under 60% oxygen, with the 0.3 μg/kg dose being most effective. It did not significantly reduce the incidence of PPCs within 7-days. The incidence of intraoperative hypoxemia was lower in the PGE_1_ groups (0/120) compared with the control group (3/40), but this was an exploratory finding as the study was not powered for this endpoint. The higher dose (0.3 μg/kg) was associated with a lower CPIS on postoperative day-2 (3.3 vs. 4.1), but CPIS is a surrogate marker, and this finding should be interpreted as exploratory. The clinical significance of this small difference is uncertain, as changes of this magnitude are below the typically recognized thresholds for meaningful clinical improvement. Hemodynamic monitoring is recommended when using higher doses of PGE_1_.

## Highlights

• Nebulized PGE_1_improves oxygenation during OLV under 60% FiO_2_.
• PGE_1_ reduces intrapulmonary shunt (Qs/Qt) without increasing PPCs.
• The 0.3 μg/kg dose provides the greatest oxygenation benefit.
• High dose PGE_1_ is associated with lower CPIS (exploratory, surrogate endpoint).
• Hemodynamic monitoring is advised for higher PGE_1_ doses.

**Abbreviations**
ASA: American Society of Anesthesiologists
BMI: Body Mass Index
Cdyn: Dynamic lung compliance
COPD: Chronic Obstructive Pulmonary Disease
CPIS: Clinical Pulmonary Infection Score
DLT: Double-Lumen endotracheal Tube
FEV_1_: Forced Expiratory Volume in one second
FiO_2_: Fraction of inspired Oxygen
FVC: Forced Vital Capacity
HR: Heart Rate
HPV: Hypoxic Pulmonary Vasoconstriction
ICU: Intensive Care Unit
MAP: Mean Arterial Pressure
NYHA: New York Heart Association
OLV: One-Lung Ventilation
PaCO_2_: Arterial Partial Pressure of Carbon Dioxide
PaO_2_: Arterial Partial Pressure of Oxygen
PEEP: Positive End-Expiratory Pressure
PGE_1_: Prostaglandin E_1_
PPCs: Postoperative Pulmonary Complications
Ppeak: Peak Airway Pressure
Qs/Qt: Intrapulmonary shunt fraction
SaO_2_: Arterial Oxygen Saturation
SpO_2_: Pulse Oxygen Saturation.

## Introduction

According to the 2020 global report on the incidence and mortality of esophageal cancer,^1^ esophageal cancer ranks eighth in incidence and sixth in mortality among all malignant neoplasms worldwide. China is a high-risk region, accounting for 53.7% of global cases and 55.3% of global deaths from this disease.^1^ Surgery is a primary treatment for esophageal cancer,^2^^,^^3^ and One-Lung Ventilation (OLV) is commonly used during these procedures. During OLV, the collapsed lung on the operative side continues to receive blood flow but is not ventilated, which increases the intrapulmonary shunt fraction (Qs/Qt) and places patients at risk of hypoxemia.^4^^,^^5^ Studies have reported that the incidence of hypoxemia during OLV ranges from approximately 5% to 10%.^6^^,^^7^

To reduce the incidence of hypoxemia during OLV, previous clinical practice widely adopted pure oxygen ventilation. However, pure oxygen inhalation can induce absorptive atelectasis, oxygen toxicity, oxidative stress, and postoperative lung injury.^8^^,^^9^ Protective lung ventilation strategies recommend using a lower Fraction of Inspired Oxygen (FiO_2_) during OLV, preferably 40%–60%, while still ensuring adequate oxygenation.^10^, ^11^, ^12^

Prostaglandin E_1_ (PGE_1_) is a selective pulmonary vasodilator. Our previous study ^13^ demonstrated that in patients undergoing esophageal cancer surgery, ultrasonic nebulization of 0.1 μg/kg PGE_1_ into the ventilated lung before OLV under 60% FiO_2_ effectively reduced Qs/Qt and improved oxygenation during the first 30-minutes of OLV. Given the significant dose-response relationship of PGE_1_, we hypothesized that different doses would exert varying effects on oxygenation and shunt reduction. Therefore, we designed this prospective randomized controlled trial to investigate the effects of different doses of ultrasonically nebulized PGE_1_ on improving oxygenation and reducing Qs/Qt under 60% FiO_2_.

## Methods

### Patients

This prospective, single-center, parallel-group, double-blind, randomized controlled trial received ethical approval from the Ethics Committee, and all procedures were performed in accordance with relevant guidelines and regulations. The study was prospectively registered with the Chinese Clinical Trial Registry (ChiCTR) on November 22, 2024 (registration n° ChiCTR2400092789). All patients were fully informed of the potential benefits and risks of participation and provided written informed consent.

After obtaining written informed consent, we recruited patients scheduled for elective esophageal cancer surgery at our hospital. Inclusion criteria were: age 65–84 years, American Society of Anesthesiologists (ASA) physical status II or III, and Body Mass Index (BMI) < 30 kg/m^2^. Exclusion criteria were: 1) History of allergy to PGE_1_, glaucoma, or gastric ulcer; 2) History of lung surgery or preoperative antitumor therapy; 3) Moderate to severe anemia; 4) Evident dyspnea or Pulse Oxygen Saturation (SpO_2_) < 90% without supplemental oxygen; 5) Presence of respiratory system infection, Chronic Obstructive Pulmonary Disease (COPD), asthma, lung infection, pleural effusion, bronchiectasis, or other lung diseases; 6) New York Heart Association (NYHA) heart failure grade III or IV, or stage 4 or higher chronic kidney disease. Additional intraoperative exclusion criteria (leading to withdrawal) were: 1) Severe arrhythmia or hemodynamic instability; 2) Operative time > 6-hours o < 2-hours; 3) Intraoperative change of surgical approach; 4) Patient or family request to withdraw from the study at any time.

### Randomization and blinding

Patients were randomly allocated into four groups: low-dose group (Group L, PGE_1_ 0.1 μg/kg), medium-dose group (Group M, PGE_1_ 0.2 μg/kg), high-dose group (Group H, PGE_1_ 0.3 μg/kg), and control group (Group C, normal saline).

On the day before surgery, a dedicated researcher invited patients to participate in the trial during a meeting in the doctor's office, in the presence of the patient, their family, and the attending physician. The researcher fully explained the potential benefits and risks of the trial, gave patients and their families sufficient time to consider, and clearly informed them that they had the right to withdraw at any time without any change in their subsequent treatment. After full communication, written informed consent was obtained.

Researcher A was responsible for patient recruitment and randomization. The random allocation sequence was generated, and the randomization numbers were sealed in an envelope together with the patient's information. The group assignments were not disclosed to other researchers before the intervention. Researcher B opened the envelope before surgery and administered the nebulized intervention according to the group assignment, but did not collect or analyze any data. Researcher C, who was blinded to group allocation and intervention, performed all data collection and statistical analysis. Unblinding was performed only after the experiment was completed. Throughout the trial, patients were unaware of their group assignment, and the researchers who collected the data were also blinded to the grouping and intervention, thereby minimizing selection bias.

### Respiratory circuit modification

To achieve unilateral lung nebulization, the atomizer was modified according to the method described in reference.^14^ The bottom of the Yuyue 402A ultrasonic nebulizer was sealed, and the two original outlets were separated and designated as the air inlet and air outlet, respectively. During mechanical ventilation with a Double-Lumen endobronchial Tube (DLT), fresh air enters through the inlet, carries nebulized drug particles through the device, and then enters the right bronchus and right lung via the outlet. The left lung (the operative side) does not receive any drug during mechanical ventilation, thus allowing the right lung to be nebulized before the start of OLV, as shown in Fig. 1, Fig. 2.

**Fig. 1:**
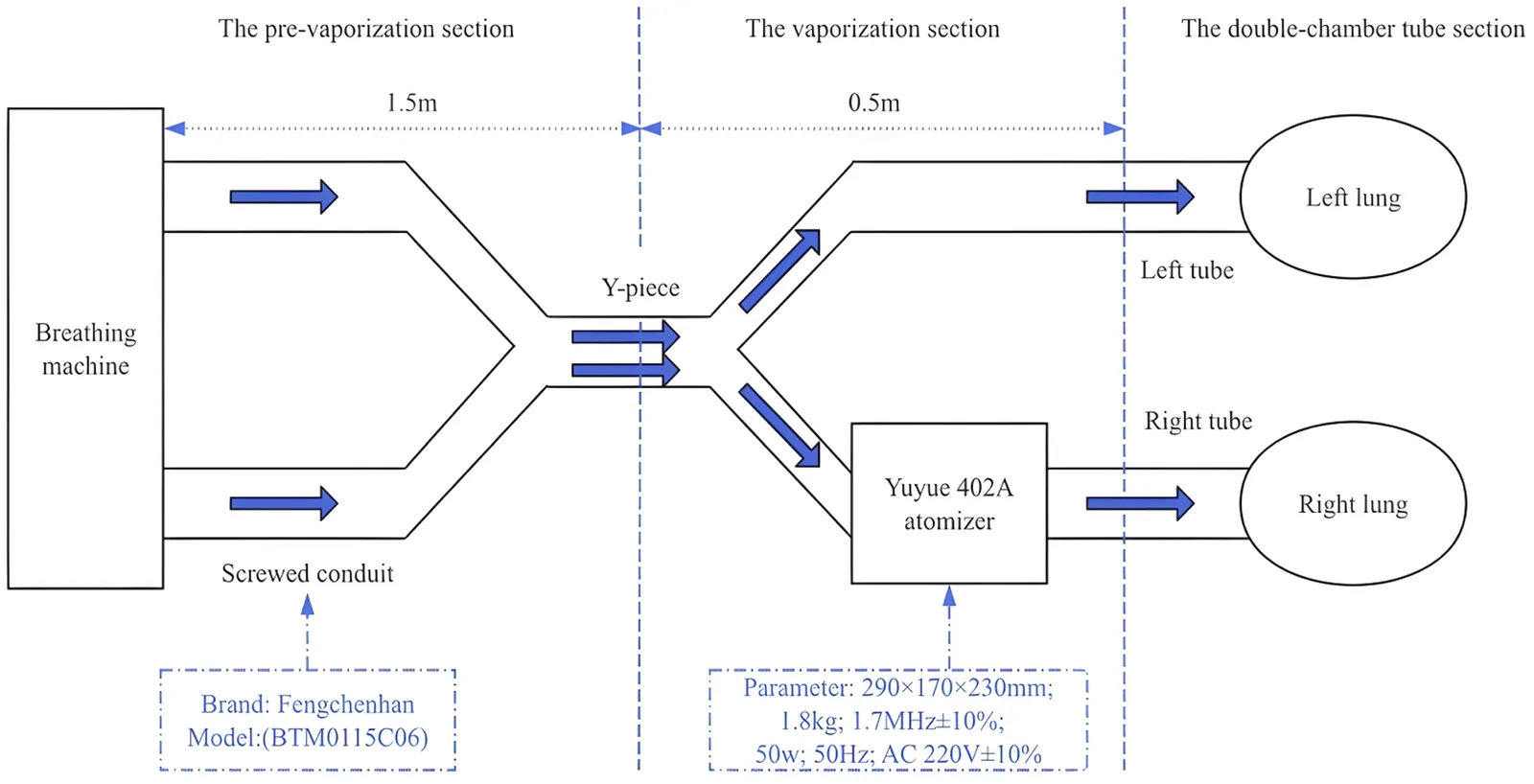
Respiratory circuit supplemental diagram.

**Fig. 2:**
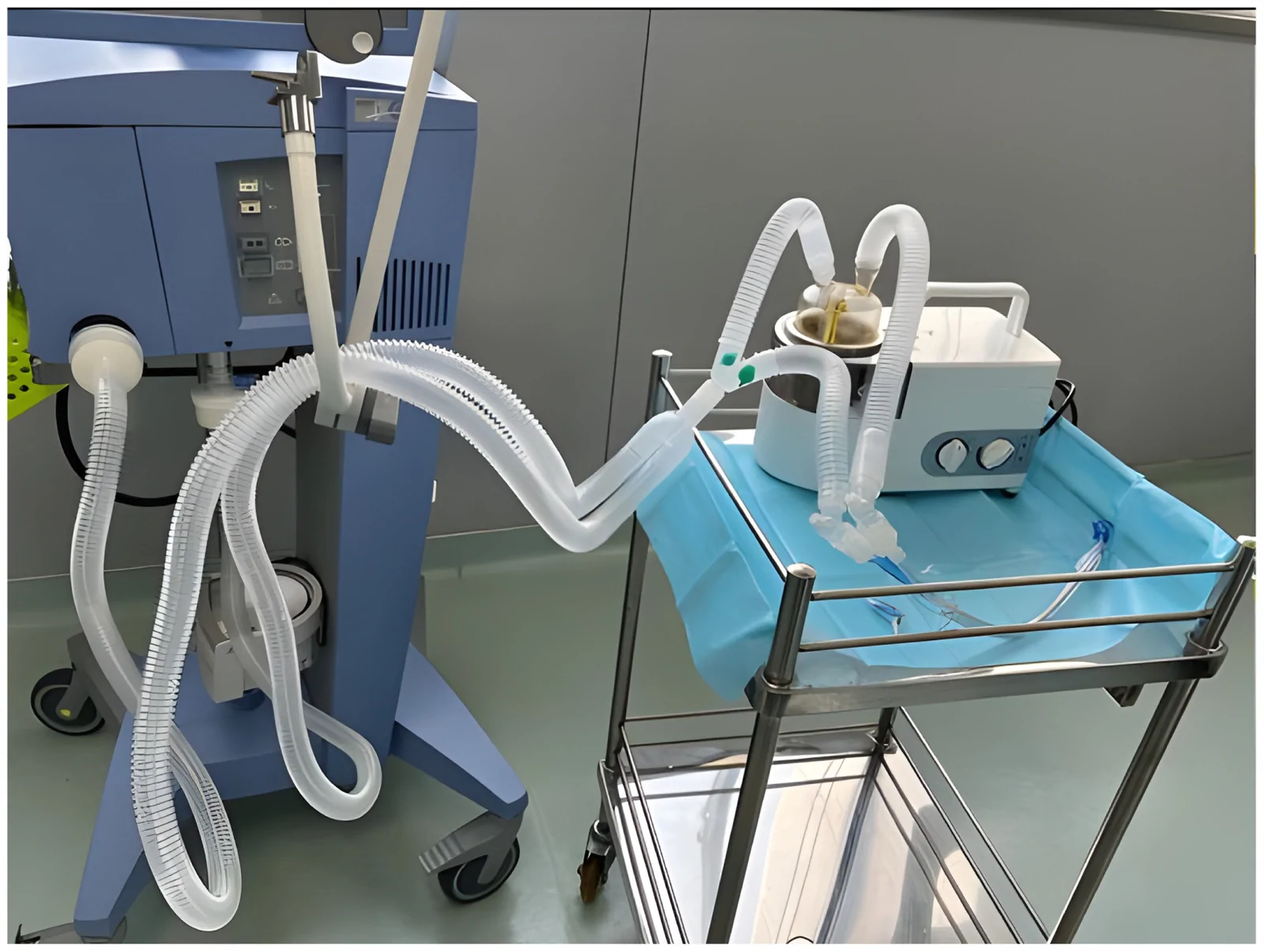
Atomizing channel structure diagram.

### Anesthesia and experimental protocol

All patients fasted for at least 8-hours and abstained from drinking for 4-hours before surgery, with no preoperative medication. Upon entering the operating room, each patient was monitored with electrocardiography, Heart Rate (HR), and SpO_2_. A right internal jugular vein catheter was inserted under ultrasound guidance, and a radial artery catheter was placed under local anesthesia for invasive arterial blood pressure monitoring.

Anesthesia was induced with midazolam 0.05 mg/kg, propofol 1 mg/kg, fentanyl 3–4 μg/kg, and rocuronium bromide 0.8 mg/kg. The appropriate size of left DLT was selected based on preoperative chest computed tomography measurements of the tracheal diameter. After full denitrogenation and oxygenation, the DLT was inserted under video laryngoscopy guidance and further positioned using a fiberoptic bronchoscope. During two-lung ventilation, the ventilation mode was set to volume-controlled ventilation with a tidal volume of 6–8 mL/kg (ideal body weight), 60% FiO_2_, an inspiratory-to-expiratory ratio of 1:2, and the respiratory rate was adjusted to maintain end-tidal CO_2_ between 35 and 45 mmHg.

With the patient in the right lateral decubitus position, the position of the DLT was readjusted using the fiberoptic bronchoscope. Subsequently, medication nebulization was administered through the modified respiratory circuit. Groups L, M, and H received 0.1, 0.2, and 0.3 μg/kg of PGE_1_, respectively, into the right lung, while the control group (Group C) received nebulized normal saline. All medications were diluted to a final volume of 10 mL before nebulization. Nebulization was performed with a gas flow rate of 2 L/min for 10 min to ensure complete delivery. After nebulization, the left lumen of the DLT was opened to the atmosphere, initiating OLV of the right lung. During OLV, tidal volume was adjusted to 4–6 mL/kg (ideal body weight), while other respiratory parameters remained unchanged.

During the maintenance phase of anesthesia, the following continuous intravenous infusions were administered: remifentanil at 0.2 μg/kg/min, propofol at 4–6 mg/kg/h, rocuronium bromide at 0.15 mg/kg/h, and dexmedetomidine at 0.2 μg/kg/h. The dexmedetomidine infusion rate was fixed at 0.2 μg/kg/h throughout maintenance in all patients and was not titrated. No sensitivity analysis by dexmedetomidine dose was performed as there was no variability in dosing. The Narcotrend index was maintained between D_2_ and E_1_. A traditional fluid management strategy was adopted, with total fluid replacement calculated as: physiological requirement + compensatory expansion volume + blood loss replacement + cumulative loss + third-space loss. Physiological requirement and cumulative loss were calculated according to the “4-2-1″ rule (4 mL/kg/h for the first 10 kg, 2 mL/kg/h for the next 10 kg, and 1 mL/kg/h for each additional kg). Compensatory expansion volume was 5 mL/kg, third-space loss was 6 mL/kg, and blood loss replacement was calculated as three times the measured blood loss.

After surgery, a sputum aspiration tube was used to clear airway secretions, two-lung ventilation was restored, and manual lung reexpansion was performed. The pressure-limiting valve was set to ensure that airway pressure did not exceed 30 cmH_2_O during reexpansion, and the reexpansion time was at least 30 s. All patients were then transferred to the Intensive Care Unit (ICU) for further monitoring and treatment. In the ICU, the ventilator was set to synchronized intermittent mandatory ventilation with a tidal volume of 6–8 mL/kg, a respiratory rate of 10 breaths/min, FiO_2_ 40%, Positive End-Expiratory Pressure (PEEP) 5 cmH_2_O, and an inspiratory-to-expiratory ratio of 1:2. After the patients regained consciousness, the DLT was evaluated by experienced physicians and removed. Extubation criteria were: 1) The patient was awake and able to respond to verbal commands; 2) Return of cough and swallowing reflexes; 3) Ability to lift the head off the pillow for >5 s; 4) Hemodynamic stability; 5) Stable breathing with a respiratory rate of 10–20 breaths/min and end-tidal CO_2_ ≤ 45 mmHg.

After surgery, all patients received intravenous patient-controlled analgesia. The analgesic pump contained sufentanil 2 μg/kg and tropisetron 0.1 mg/kg diluted with normal saline to a total volume of 100 mL. The background infusion rate was 2.0 mL/h, with a single bolus dose of 2.0 mL and a lockout interval of 15-minutes.

### Intraoperative and postoperative management

If SpO_2_ decreased below 90% and did not improve within 1-minute, hypoxemia was recorded. The position of the double-lumen endobronchial tube was immediately adjusted, airway secretions were removed, and ventilation was changed to pressure-controlled, volume-guaranteed mode with 5 cmH_2_O PEEP. If SpO_2_ continued to decrease to 85% or was not corrected within 3-minutes, the study was discontinued. Higher FiO_2_ ventilation could be used if necessary, and two-lung ventilation could be resumed to ensure patient safety during the operation.

If Mean Arterial Pressure (MAP) decreased by >20% from baseline or fell below 65 mmHg and did not improve within 1-minute, hypotension was recorded after excluding surgical causes. Phenylephrine (40 μg IV) was administered as rescue therapy, with the infusion rate accelerated and repeated doses given as needed until hemodynamic stability was restored. If MAP continued to decline to 55 mmHg or was not corrected within 3-minutes, the study was discontinued. Other vasoactive agents could be used, and continuous infusion administered, if necessary, to ensure patient safety.

### Outcomes

#### General information

We collected demographic data for each group, including sex, age, ASA classification, BMI, preoperative MAP, preoperative HR, and preoperative PaO_2_. Other baseline data included smoking index (number of cigarettes per day × years of smoking), Forced Expiratory Volume in one second (FEV_1_), Forced Vital Capacity (FVC), FEV_1_/FVC ratio, and other pulmonary function indicators. Surgical and anesthesia data included OLV duration, operation time, fluid infusion volume, blood loss, urine output, and comorbidities such as hypertension, diabetes, smoking history, and alcohol use.

#### Intraoperative data

Blood samples were collected from the radial artery and internal jugular vein for blood gas analysis at the following time points: pre-nebulization (T1), 10-min after OLV initiation (T2), 15-min (T3), 30-min (T4), 60-min (T5), and 120-min (T6). The following parameters were recorded: arterial Partial Pressure of Oxygen (PaO_2_), arterial Partial Pressure of Carbon Dioxide (PaCO_2_), Arterial Oxygen Saturation (SaO_2_), MAP, HR, Peak Airway Pressure (Ppeak), and dynamic lung compliance (Cdyn). The Qs/Qt was calculated using the following formula ^15^^,^^16^:

Qs/Qt=(CcO2−CaO2)/(CcO2−CvO)×100%

CaO2(arterialoxygencontent)=(PaO2×0.0031)+(Hb×1.34×SaO2)

CvO2(venousoxygencontent)=(PvO2×0.0031)+(Hb×1.34×SvO2)

$$\begin{array}{c} CcO2\;\left(pulmonary\;capillary\;oxygen\;content\right)\\ \;\;\;\;\;\;=\left(\left[FiO2\times \left(PB-pH2O\right)-\left(PaCO2/RQ\right)\right]\times 0.0031\right)\;\\ \;\;\;\;\;+\left(Hb\times 1.34\right),PB=Barometric\;Pressure\left(760\;mmHg\right),\;pH2O\;\\ \;\;\;\;\;=47\;mmHg,Hb=Hemoglobin,RQ\\ \;\;\;\;\;=Respiratory\;Quotient\left(0.8\right). \end{array}$$

For clinical practicality, mixed venous blood sampling via pulmonary artery catheterization was not performed, as the study population lacked clinical indications for such invasive monitoring. Instead, central venous blood was sampled from a catheter positioned in the superior vena cava near the right atrium and used as a surrogate for mixed venous blood in the calculation of CvO_2_ and Qs/Qt. This approach is commonly employed in clinical studies when pulmonary artery catheterization is not feasible.

#### Postoperative data

The Clinical Pulmonary Infection Score (CPIS) on postoperative day-2 was evaluated and recorded, with scoring criteria detailed in the literature ^17^. CPIS on postoperative day-2 was selected as a secondary endpoint because it provides an objective, composite assessment of early pulmonary infection risk (integrating clinical, radiographic, and microbiological parameters) and is a validated surrogate marker for subclinical inflammation in the postoperative period. Day-2 was chosen to capture the early inflammatory response while minimizing confounding from prolonged ventilation or later nosocomial infections.

The occurrence of Postoperative Pulmonary Complications (PPCs) within 7-days after surgery was assessed and recorded, with diagnostic criteria defined according to the European Perioperative Clinical Outcome definitions jointly issued by the European Society of Anaesthesiology and the European Society of Intensive Care Medicine in 2015.^18^

Our primary outcome measure was PaO_2_. Secondary outcome measures included the PaO_2_/FiO_2_ ratio, Qs/Qt, SaO_2_, PaCO_2_, Ppeak, Cdyn, MAP, HR, intraoperative hypoxemia, intraoperative hypotension, PPCs within 7-days, and CPIS on postoperative day-2.

### Statistical analysis

According to the preliminary experiment, the PaO_2_ values at OLV 30-min were as follows: Group L, 122±27 mmHg; Group M, 128±30 mmHg; Group H, 134±37 mmHg; and Group C, 98±21 mmHg. Sample size calculation was conducted using PASS 2021 statistical software with a two-sided significance level (α) of 0.05 and a power of 0.95. The calculated minimum sample size for each group was determined to be at least 33 patients. Considering a dropout rate of 10%–20%, a total of 40 patients were enrolled per group.

For physiological outcomes (PaO_2_, Qs/Qt, etc.), a per-protocol analysis was conducted, excluding patients with predefined protocol deviations: operative time < 2 or > 6 h, intraoperative change of surgical approach, or use of higher FiO_2_ to manage hypoxemia (which would directly affect the primary outcome). A total of 12 patients (7.5%) were excluded from this analysis. In contrast, for the secondary outcome of intraoperative hypoxemia incidence, an Intention-To-Treat (ITT) analysis was used, including all 160 randomized patients regardless of protocol adherence. This dual approach allows assessment of efficacy under ideal conditions (per-protocol) while providing an unbiased estimate of a clinically important safety event.

Data were analyzed using SPSS 20.0. Normally distributed continuous data are presented as mean ± SD and compared using one-way or repeated-measures ANOVA. Non-normally distributed continuous data are presented as median (IQR) and compared using non-parametric tests. Categorical data were compared using the Chi-Square test. A significance level of p < 0.05 was considered statistically significant. Given that the sample size was calculated based on the primary physiological outcome (PaO_2_ at OLV 30 min), the study was not specifically powered to detect differences in secondary clinical outcomes such as PPCs or the incidence of hypoxemia. These outcomes should therefore be interpreted as exploratory.

For between‑group comparisons, one‑way ANOVA was performed with a hierarchical testing strategy. Pairwise comparisons were conducted in a predefined sequence: first, each PGE_1_ Group (L, M, H) was compared with the Control Group (C) to assess the overall treatment effect; second, comparisons among the three PGE_1_ groups were performed to explore the dose‑response relationship. No adjustment for multiplicity was applied, as these comparisons were limited to prespecified contrasts directly related to the study hypotheses. The results of these comparisons should be interpreted accordingly.

The Per‑Protocol (PP) analysis served as the primary analysis for the primary outcome (PaO_2_ at OLV 30-min), as the sample size was calculated based on PP assumptions. The Intention‑To‑Treat (ITT) analysis was performed as a sensitivity analysis to assess the robustness of the findings to protocol deviations. Both analyses are reported in the Results section.

To assess the robustness of the primary outcome findings, we performed an ITT sensitivity analysis for PaO_2_ at OLV 30-min (T_4_) on all 160 randomized patients. For the three patients in the control group who required FiO_2_ escalation and were excluded from the per-protocol analysis, missing T_4_ values were imputed using the Last Observation Carried Forward (LOCF) method, i.e., their last available PaO_2_ measurements at T_3_ were used to replace the missing T_4_ values. All other patients contributed their observed data.

## Results

After screening 187 patients, 160 were randomized to the four groups (n = 40 each; Fig. 3). The final analysis included 37 patients in Group L, 37 in Group M, 38 in Group H, and 36 in Group C (Fig. 3). No significant differences were observed among the four groups in patient characteristics, surgical procedures, or anesthesia-related variables (Table 1).

**Fig. 3:**
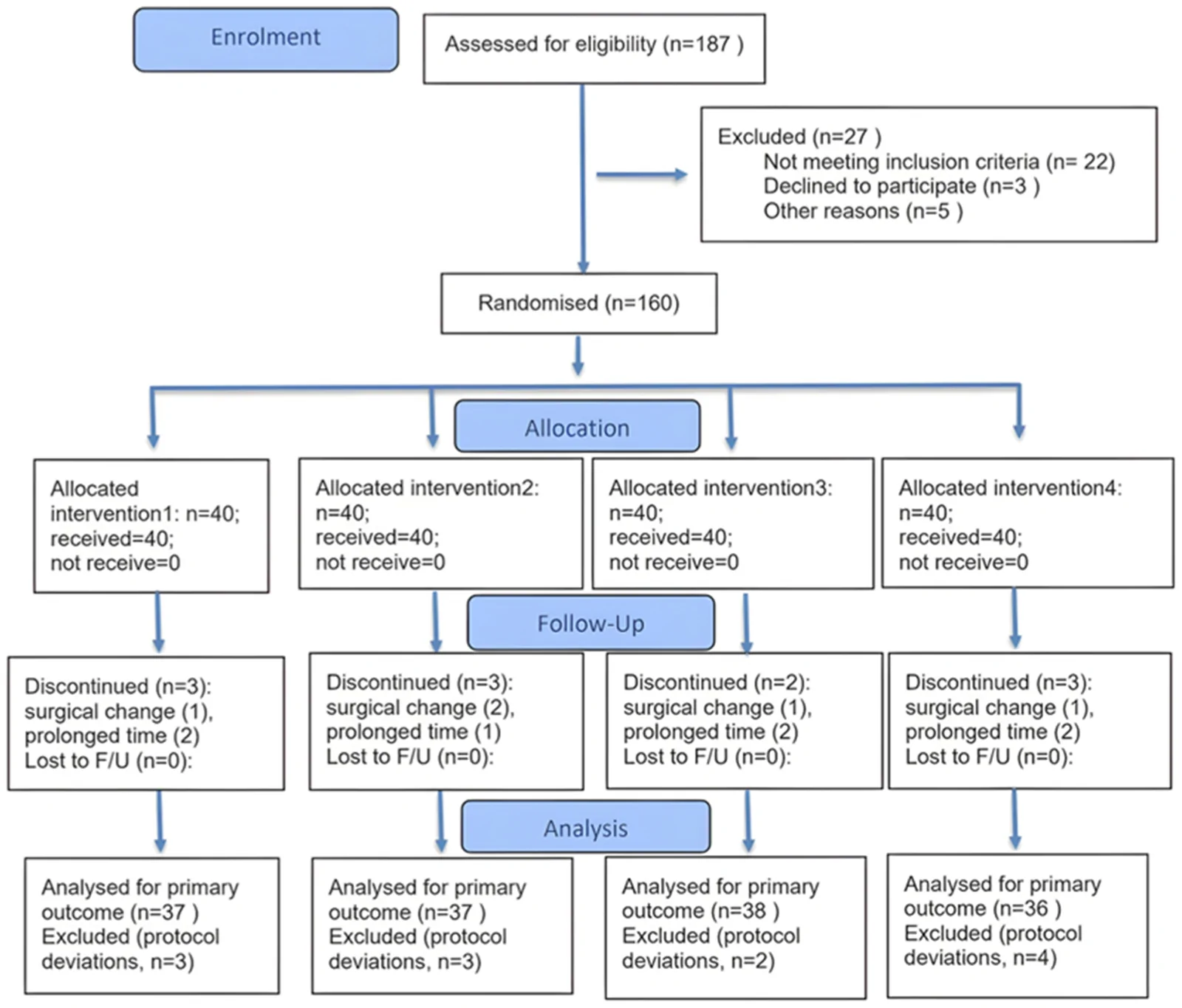
CONSORT diagram.

**Table 1:** Characteristics of patients, surgery and anesthesia.

	**Group L** **(n** **=** **37)**	**Group M** **(n** **=** **37)**	**Group H** **(n** **=** **38)**	**Group C** **(n** **=** **36)**	**p-value**
**Age (years)**	72.8 ± 8.1	72.1 ± 6.7	74.1 ± 5.7	73.6 ± 6.7	0.615
**Male, n (%)**	32(87%)	30(81%)	34(89%)	31(86%)	0.773
**ASA physical status (II/III)**	31/6	28/9	30/8	29/7	0.854
**BMI**	22.7 ± 2.4	22.7 ± 2.9	22.8 ± 2.9	22.5 ± 2.7	0.978
**Smoker, n (%)**	14(38%)	17(46%)	15(39%)	16(44%)	0.876
**Smoking index**	379±142	374±142	390±162	406±146	0.926
**FEV_1_ (L)**	3.24±0.54	3.26±0.45	3.23±0.50	3.31±0.56	0.912
**FVC (L)**	3.87±0.58	3.94±0.41	3.92±0.51	3.95±0.55	0.924
**FEV_1_/FVC ratio (%)**	83.9 ± 6.4	82.9 ± 6.5	82.4 ± 6.6	83.5 ± 6.6	0.751
**Room air PaO_2_ (mmHg)**	80.9 ± 7.7	80.4 ± 8.5	80.5 ± 8.9	79.6 ± 8.4	0.920
**Preoperative MAP (mmHg)**	93.7 ± 13.6	91.1 ± 13.9	94.5 ± 14.5	94.2 ± 17.2	0.750
**Preoperative HR (bpm)**	72.8 ± 9.7	74.1 ± 12.4	71.8 ± 13.6	76.9 ± 13.5	0.330
**Surgery duration (min)**	213±53	215±49	201±44	202±40	0.437
**OLV duration (min)**	164±37	176±39	172±43	168±32	0.526
**Anesthesia (min)**	247±57	256±50	241±45	250±53	0.676
**Infusion volume (mL)**	2027±526	2014±571	1895±370	1986±567	0.679
**Bleeding volume (mL)**	281±88	293±97	295±106	304±96	0.783
**Urine output (mL)**	318±102	347±110	351±103	346±100	0.495

Data were represented as mean ± Standard deviation (M±SD) or number of patients; ASA, American Society of Anesthesiologists; The smoking index is calculated as the number of cigarettes smoked per day multiplied by the number of smoking years; FEV_1_, Forced Expiratory Volume in one second; FVC, Forced Vital Capacity.

During the first 30-minutes of OLV, PaO_2_ and the PaO_2_/FiO_2_ ratio decreased, while Qs/Qt increased. Compared with the control group, groups L, M, and H showed significant improvements in PaO_2_, SaO_2_, and PaO_2_/FiO_2_ ratio at T_2_–T_4_, with significantly lower Qs/Qt in the three PGE_1_groups (Table 2; Fig. 4). Group H had higher PaO_2_ and PaO_2_/FiO_2_ ratio than group L at T_3_–T_4_. Compared with group H, group L had higher Qs/Qt at T_2_–T_4_, and group M had higher Qs/Qt at T_3_–T_4_ (Table 2; Fig. 4). During OLV, no statistically significant differences were observed among the four groups in PaCO_2_, Ppeak, or Cdyn (Table 2).

**Table 2:** Comparison of PaO_2,_ Qs/Qt, SaO_2_, PaCO_2_, Ppeak, Cdyn among the four groups.

	**Group**	**T_1_**	**T_2_**	**T_3_**	**T_4_**	**T_5_**	**T_6_**
**PaO_2_ (mmHg)**	L	268±39	163±44^a^	133±29^a,b^	122±27^a,b^	113±36	144±45
	M	274±41	166±45^a^	140±35^a^	126±33^a^	114±33	143±54
	H	272±45	171±45^a^	149±39^a^	137±33^a^	113±27	149±43
	C	260±58	122±31^b^	103±21^b^	97±20^b^	108±32	133±36
**Qs/Qt (%)**	L	10.9 ± 2.2	15.8 ± 2.7^a,b^	17.2 ± 1.7^a,b^	18.0 ± 1.4^a,b^	18.0 ± 1.7	16.8 ± 1.8
	M	10.7 ± 2.2	15.6 ± 2.8^a^	16.9 ± 2.1^a,b^	17.6 ± 2.0^a,b^	18.1 ± 1.8	16.8 ± 2.2
	H	11.0 ± 2.2	14.7 ± 1.9^a^	15.7 ± 1.7^a^	16.4 ± 1.4^a^	17.9 ± 1.3	16.8 ± 1.8
	C	11.2 ± 2.6	17.6 ± 1.3^b^	18.6 ± 1.3^b^	19.0 ± 1.0^b^	18.3 ± 1.6	17.4 ± 1.3
**PaCO_2_ (mmHg)**	L	42.0 ± 5.4	42.3 ± 5.8	40.6 ± 5.5	40.3 ± 5.5	42.3 ± 6.3	39.0 ± 6.4
	M	42.8 ± 5.2	42.7 ± 6.7	41.5 ± 6.5	40.6 ± 6.1	40.6 ± 5.4	39.6 ± 5.4
	H	41.4 ± 3.5	41.9 ± 5.2	41.1 ± 4.1	39.9 ± 5.0	40.3 ± 5.5	38.1 ± 5.4
	C	42.8 ± 4.2	41.7 ± 5.9	41.7 ± 5.3	41.2 ± 5.8	41.7 ± 7.0	40.9 ± 7.2
**SaO_2_ (%)**	L	99.8 ± 0.4	98.7 ± 1.1^a^	98.0 ± 1.6^a^	97.0 ± 1.8^a^	97.6 ± 1.6	98.7 ± 1.4
	M	99.9 ± 0.3	98.8 ± 1.3^a^	98.5 ± 1.3^a^	97.6 ± 1.4^a^	97.4 ± 2.1	98.3 ± 1.8
	H	99.9 ± 0.3	98.8 ± 1.1^a^	98.3 ± 1.3^a^	97.6 ± 1.8^a^	97.3 ± 1.5	98.3 ± 2.1
	C	99.8 ± 0.4	97.7 ± 1.6^b^	96.9 ± 2.3^b^	96.2 ± 2.1^b^	97.2 ± 2.1	98.3 ± 1.8
**Ppeak (cm H_2_O)**	L	15.3 ± 3.5	23.5 ± 4.1	23.6 ± 4.1	23.3 ± 4.5	23.9 ± 4.5	25.0 ± 4.4
	M	14.8 ± 2.2	23.8 ± 2.9	24.2 ± 2.9	24.5 ± 2.6	24.9 ± 3.4	25.1 ± 3.4
	H	14.8 ± 2.2	23.7 ± 3.7	24.0 ± 3.1	24.4 ± 3.6	25.1 ± 4.7	26.2 ± 4.9
	C	14.4 ± 2.4	23.9 ± 3.9	23.7 ± 3.7	23.3 ± 3.3	24.2 ± 3.8	25.2 ± 4.6
**Cdyn (mL/cm H_2_O)**	L	39.9 ± 7.7	30.3 ± 5.5	28.2 ± 4.8	27.2 ± 5.2	29.6 ± 3.4	29.9 ± 3.4
	M	39.5 ± 7.8	31.1 ± 4.0	28.0 ± 6.9	27.7 ± 4.6	29.3 ± 3.7	30.6 ± 5.5
	H	40.1 ± 6.4	30.3 ± 4.3	28.5 ± 5.4	27.6 ± 3.9	29.3 ± 5.3	30.8 ± 4.7
	C	40.5 ± 7.8	29.6 ± 4.5	28.0 ± 5.6	27.9 ± 4.6	29.6 ± 5.3	30.8 ± 5.0

Data were represented as mean ± Standard Deviation (M±SD). One-way ANOVA was used to compare PaO_2,_ Qs/Qt, SaO_2_, PaCO_2_, Ppeak, Cdyn, MAP and HR among the four groups. Group L (0.1 μg/kg PGE_1_ nebulized inhalation); Group M (0.2 μg/kg PGE_1_ nebulized inhalation); Group H (0.3 μg/kg PGE_1_ nebulized inhalation); Group C (normal saline nebulized inhalation); T_1_, pre-nebulization; T_2_, OLV 10-min; T_3_, OLV 15-min; T_4_, OLV 30-min; T_5_, OLV 60-min; T_6_, OLV 120-min.

^a^ p < 0.05 compared with Group C;.

^b^ p < 0.05 compared with Group H.

**Fig. 4:**
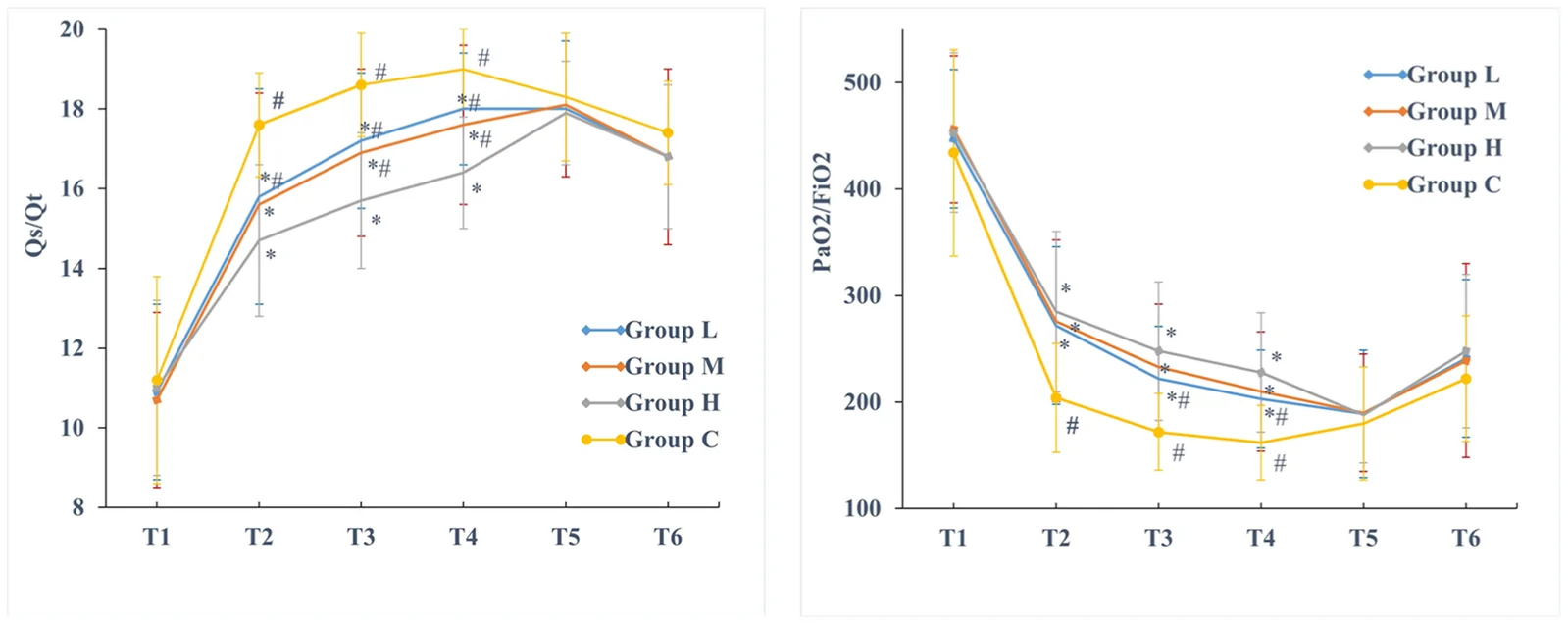
Changes in Qs/Qt and PaO_2_/FiO_2_ ratio over time during OLV. Group L (0.1 μg/kg PGE_1_ nebulized inhalation); Group M (0.2 μg/kg PGE_1_ nebulized inhalation); Group H (0.3 μg/kg PGE_1_ nebulized inhalation); Group C (normal saline nebulized inhalation); T_1_, Pre-nebulization; T_2_, OLV 10-min; T_3_, OLV 15-min; T_4_, OLV 30-min; T_5_, OLV 60-min; T_6_, OLV 120-min; * p < 0.05 compared with group C; # p < 0.05 compared with group H.

After LOCF imputation of missing T_4_ PaO_2_ values for the three control patients, the mean PaO_2_ in the control group was 97±20 mmHg. The mean PaO_2_ values were 122±27 mmHg in Group L, 126±33 mmHg in Group M, and 137±33 mmHg in Group H. The differences between each PGE_1_ group and the control group remained statistically significant (p < 0.05), and the high-dose group continued to show the highest PaO_2_ values. Thus, the primary conclusion derived from the per-protocol analysis was robust to the choice of analytical population.

The primary analysis for PaO_2_ at OLV 30 min was conducted using the PP population (n = 148). The following ITT sensitivity analysis (n = 160) was performed to test the robustness of the primary findings. In the intention-to-treat population (all 160 randomized patients), three patients in the control group developed hypoxemia that required an increase in FiO_2_ to maintain SpO_2_ ≥ 90%, whereas no patient in any PGE_1_ group required such intervention. The incidence of hypoxemia was 7.5% (3/40) in the control group versus 0% (0/120) in the combined PGE_1_ groups. However, the study was not powered for this secondary outcome; therefore, this finding should be considered exploratory and requires confirmation in a larger trial. These three patients were excluded from the per-protocol analysis for physiological outcomes because the use of higher FiO_2_ constituted a protocol deviation.

Hypotension occurred in 4 cases (10.8%) in Group L, 3 cases (8.1%) in Group M, 5 cases (13.2%) in Group H, and 3 cases (8.3%) in Group C, with no statistically significant difference in the incidence of hypotension among the four groups. Phenylephrine (40 μg IV) was administered as a rescue therapy when MAP decreased > 20% from baseline or fell below 65 mmHg for > 1-minute. The hypotension incidence therefore directly represents the proportion of patients receiving vasopressor rescue. During the first 30-minutes of OLV, all groups showed a decreasing trend in MAP (Table 3). Group H had lower MAP than Group C and Group L at T_2_, lower MAP than Group C at T_3_, and higher HR than Group C and Group L at T_3_.

**Table 3:** Comparison of MAP and HR among the four groups ($\bar{x}$ ± *s*).

	**Group**	**T_1_**	**T_2_**	**T_3_**	**T_4_**	**T_5_**	**T_6_**
MAP (mmHg)	L	99±14	97±15^b^	93±15	97±13	100±11	96±12
	M	98±16	95±13	92±12	96±14	99±12	96±11
	H	97±15	90±12^a^	88±11^a^	95±13	98±13	97±11
	C	98±13	97±13^b^	94±12^b^	97±11	100±11	98±14
HR (bpm)	L	76±12	76±12	78±12^b^	76±15	74±12	73±11
	M	76±12	77±13	81±12	79±14	76±13	72±12
	H	75±11	78±14	83±13^a^	79±11	75±11	75±10
	C	75±13	75±12	77±9^b^	77±10	74±11	71±12

Data were represented as mean ± standard deviation (M±SD). One-way ANOVA was used to compare MAP and HR among the four groups. Group L (0.1 μg/kg PGE_1_ nebulized inhalation); Group M (0.2 μg/kg PGE_1_ nebulized inhalation); group H (0.3 μg/kg PGE_1_ nebulized inhalation); Group C (normal saline nebulized inhalation); T_1_, Pre-nebulization; T_2_, OLV 10-min; T_3_, OLV 15-min; T_4_, OLV 30-min; T_5_, OLV 60-min; T_6_, OLV 120-min.

^a^p < 0.05 compared with group C; ^b^ p < 0.05 compared with group H.

Twelve patients developed PPCs within 7-days: 3 cases (8.1%) in Group L, 3 cases (8.1%) in Group M, 2 cases (5.3%) in group H, and 4 cases (11.1%) in Group C (Table 4). The overall incidence of PPCs within 7-days was 8.1%, with no statistically significant differences observed among the four groups. However, Group H was associated with a lower CPIS on postoperative day-2 compared to Group C (Table 4).

**Table 4:** PPCs and CPIS among the four groups.

	**Group L** **(n** **=** **37)**	**Group M** **(n** **=** **37)**	**Group H** **(n** **=** **38)**	**Group C** **(n** **=** **36)**
**PPCS, n (%)**	3 (8.1%)	3 (8.1%)	2 (5.3%)	4 (11.1%)
**atelectasis, n (%)**	2 (5.4%)	1 (2.7%)	1 (2.6%)	2 (5.6%)
**Pneumonia, n (%)**	1 (2.7%)	2 (5.4%)	0 (0%)	1 (2.8%)
**pleural effusion, n (%)**	0 (0%)	0 (0%)	1 (2.6%)	1 (2.8%)
**CPIS, M±SD**	3.6 ± 1.3	3.7 ± 1.4	3.3 ± 1.0^a^	4.1 ± 1.5

PPCs, postoperative pulmonary complications within 7-days; CPIS, The Clinical Pulmonary Infection Score on the postoperative second day. Data were represented as mean ± Standard Deviation (M±SD) or number of patients; the Chi-Square test was used to compare PPCs, atelectasis, Pneumonia and pleural effusion, while one-way ANOVA was used to compare CPIS among the four groups. Group L (0.1 μg/kg PGE_1_ nebulized inhalation); Group M (0.2 μg/kg PGE_1_ nebulized inhalation); Group H (0.3 μg/kg PGE_1_ nebulized inhalation); Group C (normal saline nebulized inhalation).

^a^p < 0.05 compared with Group C.

## Discussion

During OLV, hypoxemia primarily occurs because the non-ventilated lung continues to receive blood flow without ventilation, leading to an increased Qs/Qt.^19^ Therefore, optimizing the expansion and ventilation of the ventilated lung can potentially reduce shunt and alleviate hypoxemia by diverting blood from the non-ventilated lung to the ventilated lung. Our previous study demonstrated that preoperative nebulized inhalation of 0.1 μg/kg PGE1 into the right lung during OLV effectively reduced Qs/Qt and improved oxygenation.^13^ To investigate the dose-response relationship of PGE1 in reducing Qs/Qt and improving oxygenation, we designed this parallel-group controlled trial with four groups: low-dose (0.1 μg/kg nebulized PGE1), medium-dose (0.2 μg/kg), high-dose (0.3 μg/kg), and control (normal saline). Our results showed a trend toward reduced hypoxemia incidence (0/120 vs. 3/40 in the PGE1 and control groups, respectively), but this was an exploratory endpoint, and the study was not adequately powered for this comparison. In the ITT analysis, none of the 120 patients assigned to PGE1 groups required FiO_2_ escalation for hypoxemia, compared with 3 of 40 (7.5%) in the control group. Given the exploratory nature and insufficient power, these findings should be interpreted with caution and warrant validation in future adequately powered trials.

Hypoxic Pulmonary Vasoconstriction (HPV) is an automatic regulatory response of the pulmonary vasculature to alveolar hypoxia. It causes vasoconstriction in response to decreased alveolar oxygen levels, thereby redistributing blood flow within the lungs.^20^ During OLV, HPV reduces blood flow to the non-ventilated lung, optimizes the ventilation-perfusion ratio, and improves oxygenation.^21^ HPV occurs in two phases: after the initiation of OLV, PaO_2_ declines rapidly, reaching its lowest point at approximately 20–30 min, and then gradually increases over the next 1–2 h. Similar findings were observed in our study: the highest Qs/Qt values and the lowest PaO_2_ and PaO_2_/FiO_2_ ratio values all occurred at OLV 30-min. Therefore, we selected PaO_2_ at OLV 30-min as the primary outcome measure and based our sample size calculation on it. Our results demonstrated that within the first 30-minutes of OLV, different doses of PGE1 were effective in reducing Qs/Qt and improving PaO_2_ and the PaO_2_/FiO_2_ ratio. The 0.3 μg/kg nebulized PGE1 group showed the greatest improvement in oxygenation, whereas no significant differences were observed between the 0.1 μg/kg and 0.2 μg/kg groups. Our results revealed that the trends in Qs/Qt and PaO_2_ were not entirely consistent across the different dose groups. Compared with the high-dose group, the low-dose group exhibited higher Qs/Qt at OLV 10-min, with no significant difference in PaO_2_. Similarly, the medium-dose group showed higher Qs/Qt at OLV 15-min and 30-min, while PaO_2_ did not differ significantly. We speculate that this discrepancy may be attributable to dose-dependent differences in the intensity of vasodilation induced by PGE1.

Nebulized drug delivery is a topic of active clinical research because it targets the trachea and lungs directly, achieving greater efficacy with minimal systemic effects.^22^^,^^23^ Dexmedetomidine is a highly selective α_2_-adrenoceptor agonist. Intravenous administration has been shown to reduce Qs/Qt and improve PaO_2_/FiO_2_ during OLV.^24^ In the study.^22^ by Xu B et al., nebulized dexmedetomidine at an optimal dose of 2 μg/kg resulted in a Qs/Qt of 22.6 ± 2.5% and a PaO_2_/FiO_2_ of 262.7 ± 53.6 mmHg at 30-min of OLV. In contrast, in the present study, nebulized PGE_1_ at a much lower dose (0.3 μg/kg) produced a Qs/Qt of 16.4 ± 1.4% and a PaO_2_/FiO_2_ of 228±55 mmHg at the same time point. Although direct numerical comparisons are limited by differences in patient populations and study conditions, PGE_1_ achieved a lower shunt fraction and comparable oxygenation with a markedly lower dose. This difference may be explained by distinct mechanisms: dexmedetomidine acts mainly through indirect pathways such as enhancing HPV, reducing inflammation, and decreasing anesthetic requirements, whereas PGE_1_ directly dilates pulmonary arteries, a property already used clinically to treat pulmonary hypertension. A study.^23^ on nebulized iloprost (Choi H et al.) also reported favorable trends, but cross-trial comparisons are not feasible because of design heterogeneity. Direct dose comparisons are not possible due to fundamental differences in drug pharmacology, receptor targets, and mechanisms of action. The lower dose used for PGE_1_ reflects its intrinsic potency as a direct vasodilator rather than superior efficacy. Cross-trial comparisons are further limited by differences in patient populations, FiO_2_ levels, and study conditions. Therefore, head-to-head trials are required before any comparative conclusions can be drawn. Furthermore, it should be noted that all patients in the present study received a continuous background infusion of dexmedetomidine (0.2 μg/kg/h) during anesthesia maintenance. Since this infusion was administered uniformly across all groups, it is unlikely to have affected the between‑group comparisons. However, dexmedetomidine is known to enhance hypoxic pulmonary vasoconstriction, which may influence oxygenation, and thus could have attenuated or potentiated the effects of nebulized PGE_1_. Therefore, the observed oxygenation improvement cannot be attributed solely to PGE_1_, and this potential confounding should be considered when interpreting the results.

Previous studies have shown that nebulized administration has minimal effects on hemodynamics.^25^^,^^26^ Unlike these previous studies, we delivered nebulization to a single lung (the ventilated lung) during two-lung ventilation using a modified respiratory circuit. As a result, the effect of PGE_1_ was already established at the onset of OLV, which better helped patients tolerate the rapid decline in PaO_2_ during the initial 30-minutes of OLV. However, because PGE_1_ has vasodilatory properties, it may still induce hemodynamic instability. In our study, during the first 30-minutes of OLV, all groups showed a gradual decrease in MAP and a gradual increase in HR. At OLV 15-min, the high-dose group had lower MAP and HR than the control group. Nevertheless, these changes remained within 20% of preoperative values and were considered clinically acceptable. Although there was no significant difference in the incidence of intraoperative hypotension among the four groups, the 0.3 μg/kg dose of PGE_1_ still exerted a significant lowering effect on MAP at OLV 10-min and 15-min.

With the increasing adoption of Enhanced Recovery After Surgery protocols, the primary goals of thoracic anesthesia management now include not only the prevention and correction of hypoxemia but also the reduction of PPCs.^27^^,^^28^ PPCs often occur within 7-days after surgery and are associated with a mortality rate as high as 21% within 30-days,^29^^,^^30^ significantly impacting patients’ postoperative recovery and quality of life. In our study, doses of 0.1–0.3 μg/kg of PGE_1_ did not demonstrate a significant effect in reducing PPCs. While the overall incidence was lower than previously reported (8.1%vs. typical rates of 15%–30% in thoracic surgery), this may reflect the use of 60% FiO_2_ or the selected low-risk patient population rather than a PGE_1_-specific effect. Further studies are needed to clarify the role of PGE_1_ in PPC prevention.

The CPIS is commonly used to assess the severity, efficacy, and prognosis of infections. It combines clinical, radiographic, and microbiological criteria and provides significant clinical value in the diagnosis and prognostic evaluation of early-stage pulmonary infections.^31^ Fei S et al.^32^ found that PGE_1_ protects the pancreas by inhibiting the JAK2/STAT3 signaling pathway, thereby reducing inflammation and oxidative stress. Wang J et al.^33^ reported in a randomized clinical trial that PGE_1_ reduces inflammation in patients undergoing peritoneal dialysis. In our study, preoperative nebulized inhalation of 0.3 μg/kg PGE_1_ under 60% FiO_2_ was associated with a statistically significant reduction in CPIS on postoperative day-2 (3.3 ± 1.0 vs. 4.1 ± 1.5 in the control group, p < 0.001). However, CPIS is a surrogate marker, and the clinical significance of this 0.8‑point difference is uncertain. Although a generally accepted Minimal Clinically Important Difference (MCID) for CPIS has not been formally established, changes of 2–3 points are typically considered clinically meaningful in the critical care literature. The observed difference falls well below this range. Moreover, the incidence of confirmed pneumonia did not differ between groups. Therefore, this finding should be considered exploratory and hypothesis‑generating rather than definitive evidence of a lung‑protective effect.

This study has several limitations. First, the sample size was calculated based on the primary physiological outcome (PaO_2_ at OLV 30-min) and was therefore not adequately powered to detect differences in secondary clinical outcomes, including PPCs and the incidence of hypoxemia. Consequently, the lack of a statistically significant difference in PPCs among groups should be interpreted with caution, and the observed reduction in CPIS ‒ a surrogate marker rather than a confirmed diagnosis of postoperative pneumonia ‒ should be considered exploratory. Second, for the primary physiological outcomes, we used a Per-Protocol (PP) analysis as the primary analysis to avoid confounding of PaO_2_ by protocol-mandated FiO_2_ increases. We acknowledge that PP analysis may overestimate treatment efficacy by excluding patients who experienced protocol deviations, including those who required FiO_2_ escalation for hypoxemia. To address this concern, we performed an ITT sensitivity analysis with LOCF imputation for the three control patients missing T_4_ PaO_2_ values due to FiO_2_ escalation (see Results). The ITT analysis confirmed that the results remained statistically significant and consistent with the PP analysis. The three control patients excluded from PP analysis represent only 1.9% of the total sample; an ITT sensitivity analysis confirmed the primary findings remained robust. Readers should interpret the primary efficacy results with this consideration in mind. Third, the calculation of Qs/Qt relied on central venous blood as a surrogate for mixed venous blood, as pulmonary artery catheterization was not clinically indicated. While this approach is commonly accepted, it may introduce inaccuracies; therefore, the reported Qs/Qt values should be interpreted as approximate. Using superior vena cava blood rather than true mixed venous blood may lead to an underestimation of the absolute shunt fraction. Fourth, the maximum aerosol dose was set to 0.3 μg/kg based on safety considerations (daily therapeutic limit 200–400 μg), so higher doses were not explored. Although no significant difference in hypotension incidence was observed across groups, the 0.3 μg/kg dose showed transient hemodynamic effects, and its safety in patients with cardiac insufficiency remains to be determined. Fifth, only the ventilated (healthy) lung received nebulization, and blood drug concentrations were not monitored; thus, drug absorption and metabolism were not studied. Moreover, follow-up was limited to assessment of PPCs within 7-days and CPIS on postoperative day-2, without long-term prognosis or survival analysis. Sixth, the study population was strictly selected, excluding patients with COPD, asthma, obesity (BMI ≥ 30 kg/m^2^), or significant cardiopulmonary comorbidities. While this strengthened internal validity for the primary physiological outcomes, it limits the generalizability of our findings to higher-risk thoracic surgical populations. Future studies are warranted to evaluate the safety and efficacy of nebulized PGE_1_ in such patients. Seventh, the modified respiratory circuit used in this study is not a commercially available device. The circuit incorporated a Fengchenhan single-use anesthesia breathing circuit (model BTM0115C06, 1.5 m length). The Yuyue 402A ultrasonic nebulizer (1.7 MHz ±10%, 50 W, ≥ 4 mL/min, AC220V±10%, 50 Hz) was used without particle size distribution or drug deposition testing. These parameters are important for replicating the technique and ensuring consistent drug delivery. Their absence, together with the unquantified safety aspects (e.g., circuit dead space, resistance, disconnection risk), limits the reproducibility and generalizability of our findings. Consequently, the results are technique-specific and should not be extrapolated to other nebulizer systems without validation. Eighth, given the low dose (maximum 0.3 μg/kg) and aerosolized route, systemic PGE_1_ concentrations were likely very low, making clinically detectable effects such as flushing or hypotension unlikely. Nevertheless, we did not formally assess whether clinicians could identify group assignment based on physiological responses, and this potential unblinding should be considered when interpreting hemodynamic findings. However, since the primary outcome (PaO_2_) was measured from blood samples by blinded data collectors, the risk of bias for the primary endpoint is minimal. Ninth, multiple comparisons across four groups and six time points were performed without adjustment for multiplicity. However, the pairwise comparisons followed a predefined hierarchical sequence ‒ first comparing each PGE_1_ group with the control group, and then comparing among the three PGE_1_ groups. The primary conclusions are based on the first step of this hierarchy. The risk of type I error for secondary time points and exploratory analyses may be increased and should be considered when interpreting the results.

## Conclusions

Nebulized premedication with PGE_1_ at doses of 0.1–0.3 μg/kg before OLV under 60% oxygen improves oxygenation but does not reduce the incidence of PPCs within 7-days. The incidence of intraoperative hypoxemia was lower in the PGE_1_ groups (0/120) compared with the control group (3/40), but this was an exploratory finding as the study was not powered for this endpoint. The 0.3 μg/kg dose provides the greatest improvement in oxygenation and was associated with a lower CPIS on postoperative day-2; however, given the surrogate nature of CPIS, this finding should be interpreted as exploratory. Hemodynamic monitoring is recommended when using higher doses of PGE_1_.

## Consent for publication

All authors declare no conflict of interest and consent to the publication of the paper.

## Declaration of generative AI and AI-assisted technologies in the manuscript preparation process

During the preparation of this work, the authors used ChatGPT (OpenAI) for language polishing and text refinement to improve readability and clarity. After using this tool, the authors carefully reviewed and edited all content as necessary and take full responsibility for the final version of the manuscript.

## Funding

This study was funded by the Nanjing Health Science and Technology Development Special Fund Project (Grant n° YKK24252). The funder had no role in study design, data collection, analysis, interpretation, or manuscript preparation.

## Declaration of competing interest

The authors declare no conflicts of interest.
